# Health related quality of life and emergency department visits in adults of age ≥ 66 years: a prospective cohort study

**DOI:** 10.1186/s12955-018-0967-y

**Published:** 2018-07-24

**Authors:** Mahwish Naseer, Lena Dahlberg, Cecilia Fagerström

**Affiliations:** 1 School of Education, Health and Social Studies, Dalarna University, SE-791 88 Falun, Sweden; 2 Aging Research Center, Karolinska Institutet & Stockholm University, Tomtebodavägen 18A, SE-171 65 Solna, Sweden; 3 Center of Competence, Blekinge County Council, SE-371 41 Karlskrona, Sweden; 4 Health and Caring Sciences, Linnaeus University, SE 39182 Kalmar, Sweden

**Keywords:** Quality of life, Subjective health, Older adults, Care utilisation, Emergency visit

## Abstract

**Background:**

Age increases the risk of emergency department [ED] visits. Health related quality of life (HRQoL) is often estimated as an outcome of ED visits, but it can be a risk factor of ED visits. This study aims to assess the association of HRQoL with time to first ED visit and/or frequent ED use in older adults during four-year period and if this association differs in 66–80 and 80+ age groups.

**Methods:**

Data from the Swedish National Study on Aging and Care-Blekinge of wave 2007–2009 was used in combination with electronic health records on ED visits. The analytical sample included 673 participants of age 66 years and older with information on HRQoL. Cox proportional hazard model was used to assess the association between HRQoL and time to first ED visit. Logistic regression analysis was performed to estimate the association of HRQoL with frequent ED use.

**Results:**

During the study period, 55.3% of older adults visited the ED and 28.8% had a frequent ED use. Poor physical HRQoL was independently associated with first ED visit both in total sample (*p* < 0.001) and in 66–80 (p < 0.001) and 80+ (*p* = 0.038) age groups. Poor mental HRQoL had no significant association with first ED visit and frequent ED use.

**Conclusion:**

Findings suggest that poor physical HRQoL is associated with time to first ED visit in older adults. Therefore, physical HRQoL should be considered while planning interventions on the reduction of ED utilisation in older adults. Explanatory factors of frequent ED use may differ in age groups. Further studies are needed to identify associated factors of frequent ED visits in 80+ group.

## Background

Aging is a process that increases risk of functional decline and multiple health problems. In old age, fluctuations in health status from one day to another can lead to acute needs of health care. The emergency department (ED) is often an inappropriate setting for the older people with complex health problems, because of its stressful environment, long length of stay, and poor continuity of care. In Sweden, people of age 65 or more make up approximately 35% of all ED visits [1]. Frequent ED use contributes to overcrowding and longer length of stay at ED [2]. According to the Swedish National Board of Health and Welfare [3], age is an important factor for longer length of stay at ED. This points towards the importance of determining age-specific contributing factors of ED visits in order to prevent avoidable acute visits. Subjective perceptions of Health Related Quality of Life (HRQoL) can be a predictive of acute health care utilisation.

Quality of Life (QoL) is a dynamic and multidimensional concept referring to the general wellbeing of individuals. Previous research has demonstrated that health is a dominant domain of QoL according to the older people’s own definitions [4, 5], can be operationalised as the subjective perception of physical and mental health estimated from self-perceived mobility, pain, energy level, sadness, and social participation [4, 6]. A recent study on Swedish older people (65–84 years) has shown that there are stable trends in reporting poor self-rated health and severe problems in different domains of HRQoL over 8 years [7]. In clinical research, HRQoL is often measured as an outcome of a disease, intervention [8] or health care visit [9]. Poradzisz and Florczak, [8] argue that HRQoL could influence adherence to medication, healthy life style and self-management. Therefore, instead of seeing HRQoL only as a designated outcome, future research should consider HRQoL as an input. This study will focus on whether HRQoL is associated with time to first ED visit and/or frequent ED use.

Previous studies have shown that self-rated health and HRQoL are important predictors of ED visit [10–12] and frequent ED use [2]. However, these studies have focused on people with specific health problems, have sampled from a broad age range [10, 11], or have included outpatients of geriatric centre or people from particular settings, such as older people living in their own homes [12]. Focusing on specific settings or health problems may only help to reduce ED visits for particular group and using samples that have a broad age range may mask the influence of age specific characteristics such as living circumstances, high risk of multi-morbidity or poor HRQoL [7]. Therefore, to reduce ED visits it is essential to determine age specific risk factors of ED and consider frequent utilization of ED [2]. However, there is a lack of studies on HRQoL and ED visits based on populations including both people living in their own homes and in institutions of older adults and analysing separate age groups. Moreover, previous research has shown that sociodemographic variables such as age, gender, education, housing, health care use [13], health and functional status [12, 13] and living alone [14] can explain ED visits in older people. Therefore, it is important to consider these explanatory variables while exploring the association between HRQoL and ED visits.

The aim of this study was to investigate if HRQoL is associated with time to first ED visit and/or frequent ED use in older adults during a 4 year-period. A second aim was to examine whether this association differs between the 66–80 and 80+ age groups.

## Methods

### Study design, population and procedure

This study has a prospective cohort design. It is based on the Swedish National Study on Aging and Care (SNAC), which is an ongoing longitudinal multicentre cohort study on adults of age 60 years and more, with its first data collection wave in 2001–2003. Data was collected via self-administrated questionnaires. In SNAC, participants are followed up every sixth years for < 80 and every third year for 80+. The following age cohorts are included 60, 66, 72, 78, 81, 84, 87, 90, 93, and 96 years. For further details of SNAC, see [15].

In this study, data from one out of four sites of SNAC, that is, SNAC-B (Blekinge), is used. SNAC-B covers a community of Sweden with approximately 62,300 inhabitants. In SNAC-B, national population register was used to invite the subjects for participation in study. For age cohorts 60, 66, 72 and 78, people were selected randomly, while the entire population was invited to participate in the study for age cohorts 81, 84, 87, 90, 93 and 96 years. In the SNAC-B of wave 2007–2009, 978 subjects of age ≥ 66 years were invited to participate in the study. The subjects of age cohort 60 years were not included in this wave as it was the first follow up after 6 years of those who participated in the wave 2001–2003 of SNAC-B. Of 978 invited participants, 841 (85.9%) agreed to participate in the study, 63 refused to participate, 46 received the invitation but died before data collection, and other reasons of non-participation were poor health (*n* = 12), moved to another region of Sweden (*n* = 10) or not being reachable (*n* = 6).

In the present study, data from the SNAC-B of wave 2007–2009 was used as baseline. The inclusion criteria used in this study were that the participants should be 66 years of older and have provided information on HRQoL, which gave an analytical sample of 673 participants of age ≥ 66 years including individuals living in their own homes and institutionalised housing. Electronic health care record was used to obtain information on ED visits during 4 years from baseline. No difference was observed for ED visits for who did or did not provide information on HRQoL. However, individuals with missing information on HRQoL were older, more dependent in their activities of daily living (ADL) and their instrumental activities of daily living (IADL), and they more likely to live in institutions, live alone and have diseases/illnesses.

Data was collected in accordance to the Helsinki declaration, and both verbal and written informed consent was obtained. The SNAC-B in combination with electronic health record has been awarded ethical approval by the ethics committee of Lund University (LU 128–00, LU 604–00).

### Materials

In this study, time to first ED visit and frequent ED use served as dependent variables in the analyses. Information on ED visits was obtained from electronic health record held by the Blekinge County Hospital of Sweden, and was dichotomised as having/not having at least one ED visit during 4 years. In previous research, a varying number of visits (≥4 - ≥6) over different time periods have been used to define frequent ED use [2, 16, 17]. In this study, frequent ED use was defined as having four or more ED visits during a 4 year-period.

HRQoL was measured at baseline via Short-Form Health Survey (SF-12), collected through the questionnaire. SF-12 is a validated instrument [6] and is widely used in older adults. It consists of two dimensions: physical and mental. Physical HRQoL includes subjective perception of general health, difficulty in performing moderate activities, climbing of several flights of stairs, bodily pain, and accomplishing less due to physical health, while mental HRQOL includes not being careful in daily activities, accomplishing less due to mental health, social interaction, energy level, sadness, calmness, and peacefulness [6]. Sullivan’s algorithm was used to compute the SF-12 score [18]. The score of each dimension of SF-12 ranges from 0 (poor) to 100 (good) HRQoL. There is no gold standard definition of poor HRQoL. Lowest quartile of HRQoL score was used to define the poor HRQoL [19].

Information on other independent variables was collected through the questionnaire. Dichotomous variables were used for gender (male/female), housing (living in their own home/institutionalised housing), living arrangement (living alone/together with somebody), and education (primary school education/higher education). A continuous variable was constructed for number of diseases/illnesses (cardiovascular, diabetes, cancer, arthritis, depression, dementia, fracture, osteoporosis, thyroid, tuberculosis, Parkinson’s disease, asthma, trauma, inflammation, epilepsy, chronic obstructive pulmonary disease, bipolar, cataract, sleep apnea, and snoring). Score “1” was given for each disease, ranging from 0 (no disease) to score 20. Katz index was used to measure the activities of daily living (ADL; bathing, dressing, toileting, transferring, continence and feeding) [20], and instrumental activities of daily living (IADL; cleaning, transportation, shopping and cooking) [21]. The score “1” was given for each dependency with score range 0–6 for ADL and 0–4 for IADL. Functional dependence was defined as ADL ≥1 and IADL ≥1, respectively.

### Statistical analysis

As broad age ranges may mask the influence of changes in the demographic characteristics and health status of study population with age, the study sample was stratified into two age groups: 66–80 years and 80+ years. To make comparison between age groups Chi-squared test was used for binary data and t-test for interval data. Due to the significant differences in the characteristics of two age groups (see Table 1) further analysis were conducted both for the whole group and separately for each age group. To estimate the association of HRQoL with ED visits, dichotomous variables were constructed for physical and mental HRQoL. The score in the lowest quartile was defined as poor HRQoL. The cut-offs ≤33.2 and ≤ 50.1were used to define poor physical and mental HRQoL, respectively. Time to event data was computed from the date of baseline data collection to the date of first ED visit during 4 year-period. Time from baseline to mortality and/or end of follow-up (31 December 2011) was used as censored.

**Table 1:** Characteristics of the total sample and stratified by age groups 66–80 years and 80+ years

Variables	Total sample (*n* = 673)	Age 66–80 (*n* = 445)	Age 80+ (*n* = 228)	*p*-value
Physical HRQoL mean (SD)	43.0 (11.7)	45.8 (10.5)	37.5 (11.8)	*< 0.001* *< 0.001*
Poor %	25.0	16.4	41.7	
Good %	75.0	83.6	58.3	
Mental HRQoL mean (SD)	53.8 (8.5)	54.7 (7.9)	52.1 (9.4)	*< 0.001* *< 0.001*
Poor %	25.0	20.0	34.6	
Good %	75.0	80.0	65.4	
Male %	43.4	44.5	41.2	0.418
Institutionalised housing %	4.3	1.6	9.6	*< 0.001*
Living alone %	40.4	28.1	64.5	*< 0.001*
Primary education %	48.9	43.8	59.6	*0.001*
Dependent in ADL (≥1, %)	9.8	5.2	18.9	*< 0.001*
Dependent in IADL (≥1, %)	57.6	48.5	75.3	*< 0.001*
Number of diseases/illnesses mean (SD)	1.4 (1.2)	1.4 (1.2)	1.2 (1.0)	*0.048*
ED visit (yes, %)	55.3	47.9	69.7	*< 0.001*
Frequent ED use (yes, %)	28.8	27.2	30.8	0.450

*Abbreviations*: *HRQoL* Health related quality of life, *SD* standard deviation, *ADL* activity of daily living, *IADL* instrumental activity of daily living, *ED* emergency department. Dichotomous variables were constructed for physical and mental HRQoL. Lowest quartile with cut-offs ≤33.2 and ≤ 50.1 were used to define poor physical and mental HRQoL, respectively. Twenty different diseases/illnesses were included in the variables number of diseases/illnesses (range: 0–6). The internal dropout for variable education was 22% for 66–80 and 27% for 80+ group. The Chi-squared test was used for nominal data and t-test for interval data to compare the difference between the age groups

Significance is tested as *p* < 0.05 are captured in italic

Cox-proportional hazard model [22] was used to test the association of physical and mental HRQoL with time to first ED visit. To avoid association by chance, physical and mental HRQoL variables were entered with all independent variables simultaneously at the first step of Cox proportional model and backward Likelihood Ratio (LR) method was used. The LR accounts for likelihood of a variable to satisfactorily explain outcome variable [23]. Default setting of probability for stepwise entry, that is, 0.05 entry and 0.10 for removal was used. The results are presented as hazard ratio (HR) with 95% confidence interval (CI).

Logistic regression was used to test the association of physical and mental HRQoL with frequent ≥4 ED visits. In the logistic regression models, all variables were entered simultaneously and backward LR method was used. The results are presented as odds ratio (OR) with 95% CI.

In order to analyse the stability (internal validation) of final models in the Cox and Logistic regression bootstrap approach with 1000 bootstrap samples (default settings) was performed [24]. In the bootstrap, simple sampling method with bias-corrected and accelerated confidence interval type were used. To test significance, *p* < 0.05 was used. SPSS version 24 for Windows was used to conduct the analysis.

## Results

### Descriptive analyses

At baseline, the mean age of the participants was 76.8 years and 43.4% of them were men. The mean scores of physical and mental HRQoL were significantly lower in the 80+ age group compared to the 66–80 age group, and the percentages of participants with poor physical and mental HRQoL were higher in the 80+ group than 66–80 group (*p* < .001) (Table 1). In addition, compared to the 66–80 age group, individuals in the 80+ group were more likely to live in institutionalised housing (*p* < 0.001), live alone (*p* < 0.001), have primary school education (*p* = 0.001), and be dependent in ADL and IADL (*p* < .001). However, the mean number of diseases/illnesses was higher in the 66–80 group than 80+ group (*p* = .048). During the 4 year-period, 55.3% of the total sample had at least one ED visit and 28.8% had frequent ED visits. The percentage of individuals having an ED visit was higher in the 80+ group than 66–80 years group (*p* < .001). No significant difference between the age groups were observed regarding frequent ED use.

**Fig. 1:**
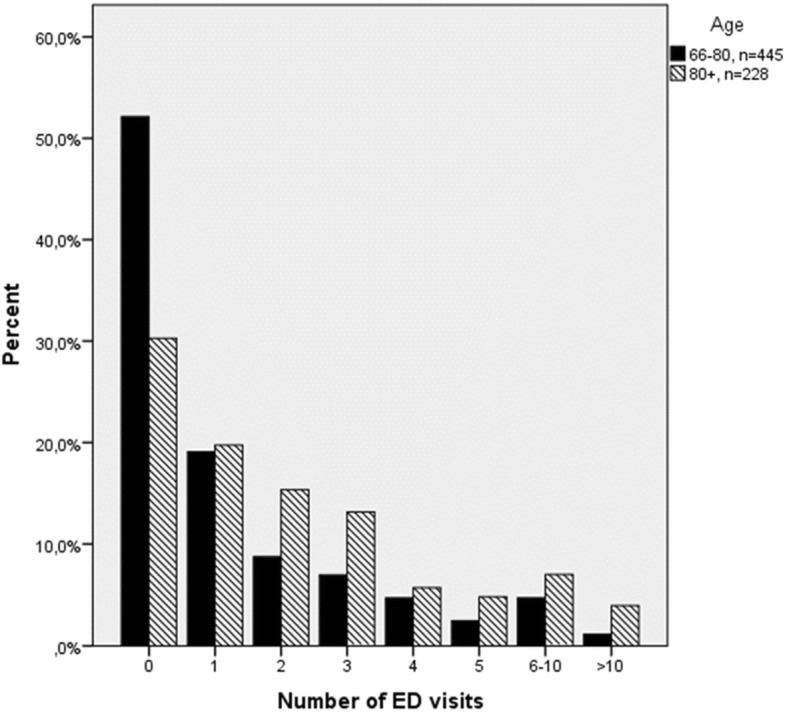
Number of emergency department visits during the study period in 66–80 and 80+ age group

The median follow-up period was 2.77 years (range 0.01 to 4.42 years). Compared to the 66–80 age group, people aged 80+ were more likely to have one or more ED visits during the study period (Fig. 1).

### Multivariate analyses of time to first ED visit

Poor physical HRQoL showed 68% (HR 1.68, 95% CI 1.29–2.20) increased hazard of time to first ED visit compared to good physical HRQoL, in the total study population (Table 2, final model). Association between poor physical HRQoL and time to first ED was also significant in 66–80 (HR 1.97, 95% CI 1.34–2.88) and 80+ (HR 1.46, 95% CI 1.02–2.10) age group. However, no significant association between poor mental HRQoL and time to first ED visit was observed.

**Table 2:** Cox proportional hazard regression backward likelihood ratio (LR) model for first emergency department (ED) visit

Variables	Total (*n* = 503)		66-80 years (*n* = 339)		80+ years (*n* = 164)	
	HR (95% CI)	*p*-value	HR (95% CI)	*p*-value	HR (95% CI)	*p*-value
Model step one^a^						
Poor Physical HRQoL	1.53 (1.12-2.10)	*0.007*	1.97 (1.28-3.02)	0.002	1.22 (0.77-1.91)	0.387
Poor Mental HRQoL	1.13 (0.85-1.51)	0.391	1.11 (0.74-1.67)	0.592	1.13 (0.75-1.70)	0.544
Age (80+)	1.71 (1.31-2.24)	*<0.001*	-	-	-	-
Male	1.18 (0.91-1.52)	0.205	1.11 (0.80-1.54)	0.500	1.29 (0.82-2.03)	0.262
Institutionalised housing	0.90 (0.49-1.64)	0.738	1.44 (0.50-4.12)	0.487	0.74 (0.35-1.55)	0.427
Living alone	1.10 (0.83-1.46)	0.489	1.06 (0.74-1.53)	0.732	1.29 (0.79-2.11)	0.295
Primary education	1.16 (0.91-1.47)	0.228	1.06 (0.77-1.45)	0.713	1.34 (0.90-1.99)	0.147
Dependent in ADL (≥1)	1.04 (0.68-1.57)	0.852	0.80 (0.41-1.58)	0.536	1.14 (0.67-1.94)	0.627
Dependent in IADL (≥1)	1.21 (0.93-1.57)	0.143	1.16 (0.83-1.61)	0.376	1.34 (0.84-2.14)	0.215
Number of diseases/illnesses	1.08 (0.98-1.19)	0.104	1.14 (1.01-1.29)	*0.026*	1.02 (0.86-1.20)	0.785
Final model^b^						
Poor Physical HRQoL	1.68 (1.29-2.20)	<0.001*	1.97 (1.34-2.88)	<0.001*	1.46 (1.02-2.10)	0.038*
Age 80+	1.74 (1.34-2.25)	<0.001*	-	-	-	-
Dependent in IADL	1.27 (0.98-1.63)	0.067	-	-	-	-
Number of diseasees/illnesses			1.15 (1.02-1.29)	0.021*	-	-

The sample only includes respondents who have responded to all included covariates and is, thus, smaller than in Table 1. *Abbreviations*: *HRQoL* Health related quality of life, *ADL*, activity of daily living, *IADL* instrumental activity of daily living, *HR* hazard ratio, *CI* confidence interval. In the analysis, good physical and mental health related quality of life, female, living at home, living together, education higher than primary, independence in ADL and IADL, and having no disease/illness were used as reference categories. The *p*-value < 0.05 was used to test significance. ^a^Cox proportional hazard regression backward LR at step one including all variables. ^b^Cox proportional hazard regression backward LR at final step. In total sample, LR at step one was 3167.37, *p* < 0.001 and at final step was 3174.23, *p* < 0.001. In 66–80 age group, LR at step one was 1725.29, *p* = 0.004 and at final step was 1728.33, *p* < 0.001. In 80+ age group, LR at step one was 1058.87, *p* = 0.214 and at final step was 1066.54, *p* = 0.037

*Significant *p*-value based on 1000 Bootstrap samples with bias-corrected and accelerated confidence interval type

Moreover, age above 80 was significantly associated with time to first ED visit (HR 1.74, 95% CI 1.34–2.25) in the total study sample. For the 66–80 age group, people with diseases/illnesses (HR 1.15, 95% CI 1.02–1.29) exhibited increased likelihoods of time to first ED visit, but not in the 80+ age group.

### Multivariate analyses of frequent ED use

In the total study population, poor mental HRQoL was significantly associated (OR 1.88, 95% CI 1.07–3.32) with frequent ED use compared to good mental HRQoL (Table 3, final model). However, this association no longer remained significant after the robustness analysis done by bootstrap approach. For frequent ED use, physical HRQoL was not significant. In the 66–80 age group, people living in institutionalised housing (OR 11.69, 95% CI 1.04–131.62), having primary education only (OR 2.32, 95% CI 1.10–4.88), and being dependent in ADL (OR 3.68, 95% CI 1.01–13.29) were more likely to have frequent ED use.

**Table 3:** Logistic regression backward likelihood ratio (LR) model for frequent emergency department (ED ≥ 4) use among respondents who have had a first ED visit

Variables	Total (*n* = 280)		66-80 years (*n* = 161)		80+ years (*n* = 119)	
	OR (95% CI)	*p*-value	OR (95% CI)	*p*-value	OR (95% CI)	*p*-value
Model step one^a^						
Poor Physical HRQoL	1.38 (0.73-2.60)	0.317	1.04 (0.37-2.86)	0.938	1.23 (0.50-3.00)	0.649
Poor Mental HRQoL	1.64 (0.89-3.03)	0.108	1.42 (0.56-3.56)	0.450	1.70 (0.69-4.17)	0.242
Age (80+)	0.72 (0.38-1.34)	0.308	-	-	-	-
Male	1.77 (1.01-3.11)	*0.045*	2.10 (0.97-4.56)	0.059	1.67 (0.68-4.13)	0.260
Institutionalised housing	1.34 (0.39-4.60)	0.635	10.36 (0.83-128.32)	0.069	0.76 (0.13-4.29)	0.759
Living alone	1.47 (0.81-2.66)	0.199	1.22 (0.55-2.74)	0.615	1.76 (0.64-4.83)	0.273
Primary education	1.32 (0.75-2.29)	0.326	2.33 (1.10-4.94)	0.026	0.68 (0.28-1.62)	0.389
Dependent in ADL (≥1)	0.88 (0.36-2.14)	0.784	3.53 (0.69-17.93)	0.128	0.46 (0.14-1.53)	0.211
Dependent in IADL (≥1)	0.98 (0.53-1.79)	0.952	0.81 (0.37-1.74)	0.594	1.30 (0.44-3.89)	0.628
Number of diseases/illnesses	1.00 (0.81-1.24)	0.935	0.92 (0.70-1.22)	0.589	1.05 (0.72-1.53)	0.800
Final model^b^						
Poor Mental HRQoL	1.88 (1.07-3.32)	*0.028*	-	-	1.69 (0.74-3.83)	0.205
Male	1.56 (0.92-2.65)	0.099	1.92 (0.92-4.04)	0.082	-	-
Institutionalised housing	-	-	11.69 (1.04-131.62)	0.046*	-	-
Primary education	-	-	2.32 (1.10-4.88)	0.025*	-	-
Dependence in ADL	-	-	3.68 (1.01-13.29)	0.047*	-	-

*Abbreviations*: *HRQoL* Health related quality of life, *ADL* activity of daily living, *IADL* instrumental activity of daily living, *OR* odds ratio, *CI* confidence interval. In the analysis, good physical and mental health related quality of life, female, living at home, living together, education higher than primary, independence in ADL and IADL, and having no disease were used as reference categories. The *p*-value < 0.05 was used to test significance

^a^Logistic regression backward LR at step one including all variables

^b^Logistic regression backward LR at final step

In total sample, LR at step one was 323.63, *p* = 0.04 and at final step was 328.10, *p* = 0.024. In 66–80 age group, LR at step one was 175.52, *p* = 0.101 and at final step was 177.05, *p* = 0.092. In 80+ age group, LR at step one was 135.97, *p* = 0.052 and at final step was 140.79, *p* = 0.013

*Significant *p*-value based on 1000 Bootstrap samples with bias-corrected and accelerated confidence interval type

## Discussion

The aim of this study was to determine whether HRQoL is associated with time to first ED visit and/or frequent ED use in older people and if this association differs between the 66–80 and 80+ age groups. Fifty five percent of the total sample had at least one ED visit during 4 year-period and twenty eight percent had frequent ED use. Poor physical HRQoL was significantly associated with the time to first ED visit both in the total sample and in both age groups. For frequent ED use, associations with poor physical and mental HRQoL were not significant. Other significant factors of time to first ED visit were age above 80 in total sample and number of diseases/illnesses in 66–80 age group. Institutionalised housing, primary education, and ADL dependence were significantly associated with frequent ED use in 66–80 age group, however not in 80+ group.

Age may not have a direct correlation with time to first ED visit, but the risk of health problems associated with aging increase the risk of an ED visit. In this study, the characteristics of the 80+ age group can explain the association between the 80+ age category and time to first ED visit. Risk factors such as poor perceived health and aging may have some similarities with number of diseases, ADL and IADL dependence [25]. In our study, this may have masked the effect of other variables, such as IADL. Therefore, different models were tried by excluding HRQoL, ADL and IADL (not shown here). Exclusion of HRQoL improved the effect of IADL and number of diseases/illnesses while exclusion of ADL and IADL did not effect on the significance of HRQoL.

The association between poor physical HRQoL and time to first ED visit both in the total sample and age groups are in line with the findings of previous research [10, 12]. A mixed method study [4] showed that physical health was the most important domain of QoL for older people and that this might be due to the fact that poor physical health impacts on mobility, which ultimately leads to dependence. The measurement of physical HRQoL is broader and includes not only subjective assessments of health but also the impact of subjective health on individual’s mobility and independence. This may explain the association between physical HRQoL and time to first ED visit in present study. Findings suggest that improvement in physical HRQoL can contribute to decrease the ED utilization in older people. Previous research suggests that preservation of mobility and/or functional ability and health promotion counselling can positively contribute to self-perceived health [9, 25].

In the present study, perhaps surprisingly the number of diseases/illnesses showed increased likelihood for ED visits in 66–80 but not in the 80+ group. This can be explained by the characteristics of the study sample, that is, that the 80+ group had a lower mean number of diseases/illnesses than the 66–80 group and by “selective mortality”. Participants with more health problems may have not reached to the age of 80+ or did not participate in the study because of their health status [15].

Poor mental HRQoL was not significant for the time to first ED visit and/or for frequent ED use. Lack of studies on the association of mental HRQoL and ED visits limit the comparison and explanation of this finding in relation to previous research. However, this finding contradicts a Norwegian study on hospital admissions in nursing home residents [26]. One explanation could be the different outcomes, that is, that hospital admissions that can be either planned or unplanned while ED visits are always unplanned. Another explanation to the contradictory results could be that the study population in the Norwegian study was limited to nursing home residents [26].

Institutionalised housing, primary school education and dependence in ADL were significant risk factors of frequent ED use in 66–80 age group. However, variables included in this study did not explain frequent ED use in 80+ group. Age is often considered as a control variable in the studies on ED visits [2, 12]. This limits the ability to compare with previous research and find explanations to why some variables were important for below 80 age group but not in 80+. Thus, future studies are warranted to explore the risk factors of frequent ED use in 80+ group.

### Strengths and limitations of the study

There are few potential limitations that should be considered while interpreting findings of this study. HRQoL is a subjective health measure that is dynamic in nature, but it is only measured at one time point in this study. Moreover, aging is not a state but a process associated with health problems, and the severity of these health conditions can vary over time, particularly in the 80+ age group. This might have influenced the findings of this study. A limitation of this study is that self-reported information on independent variables such as diseases/illnesses might be influenced by recall bias. Moreover, this study was based on a regional sample of older adults and, considering organisational differences across regions (and countries), results may not be generalised to the whole population. In addition, participants who provided information on HRQoL were younger, healthier with fewer diseases/illnesses (mean ≈ 1.4), better functional ability, and more likely to live in their own home together with relatives than those who did not respond to the HRQoL item. However, no difference was observed for ED visits between those who did or did not provide information on HRQoL. In this study, population with cognitive impairment was underrepresented. Current and previous health care use could explain current ED use [13], but this was not considered in this study due to unavailability of information.

A strength of this study is that it is based on a population based sample rather than a clinical sample, and that it includes older adults living in their own home and in institutionalised housing. Another strength is that information on ED visits was obtained from hospital records, that is, recall bias did not influence the outcome variables of this study. A prospective study design with a long follow-up period and separate analyses for different age groups are other strengths of this study.

## Conclusion

In this study, 55.3% of older adults visited the ED and 28.8% had a frequent ED use during 4 year-period. This study identified different explanatory variables of ED visits specific to each age group. This highlights the importance of age stratified analyses. However, some similarities were also observed, for example poor physical HRQoL increased the likelihood of first ED visit in both age groups.

Poor physical HRQoL was associated with increased hazard of time to first ED visit independent of diseases/illnesses, both in total sample and in age groups. Findings suggest that improvement of physical HRQoL in adults of age ≥ 66 years has the potential to reduce the first ED visit. Health promotion counselling in other settings than EDs, such as geriatric centres or primary health care, could help to identify and address risk factors behind poor HRQoL. Ultimately, this could contribute in the decline of acute care utilization. Risk factors of frequent ED use in 80+ group are still unclear, and future studies on this age group is needed.

## Abbreviations

ADL: Activity of Daily Living
CI: Confidence Interval
ED: Emergency Department
HR: Hazards Ratio
HRQoL: Health Related Quality of Life
IADL: Instrumental Activity of Daily Living
OR: Odds Ratio
SD: Standard Deviation
SF: Short Form
SNAC-B: The Swedish National Study on Aging and Care-Blekinge
SPSS: Statistical Package for Social Science
